# Two-point fixation enhanced the outcome of laparoscopy-assisted ventriculoperitoneal shunt in adult patients with hydrocephalus: a retrospective study

**DOI:** 10.3389/fsurg.2023.1135818

**Published:** 2023-07-13

**Authors:** Jing-Nan Wu, Yu-Jie Zhou, Lei Wang, Jin-Lu Gan, Jian Wang, Hong-Yang Zhao, De-Qiang Lei

**Affiliations:** Department of Neurosurgery, Union Hospital, Tongji Medical College, Huazhong University of Science and Technology, Wuhan, China

**Keywords:** hydrocephalus, ventriculoperitoneal shunt, laparoscopy-assisted ventriculoperitoneal shunt, two-point fixation, shunt survival, liver ligaments

## Abstract

**Objective:**

In patients with hydrocephalus, laparoscopy significantly improved ventriculoperitoneal shunt (VPS) outcomes. However, abdominal complications still occur, which require revision surgeries. In this study, we aimed to examine whether laparoscopy-assisted VPS with two-point fixation (LAVPS-TPF) has better outcomes than those of VPS (open-VPS) and laparoscopy-assisted VPS with no fixation (LAVPS-NF).

**Methods:**

We retrospectively reviewed clinical records of 105 open-VPS, 40 LAVPS-NF, and 49 LAVPS-TPF cases from 2015 to 2020. Data including body mass index, etiology, abdominal surgery history, Glasgow coma scale (GCS), operation time, in-hospital days, shunt failure, complications, and modified Rankin scores were analyzed, as well as subgroups of patients with history of abdominal surgery, GCS scores, and revision surgeries.

**Results:**

The LAVPS-TPF group demonstrated decreased shunt failure rates at 12 months (2.04%) compared to those of the open-VPS group (14.29%, *P *= 0.020) and reduced abdominal shunt-related complications (*P *= 0.004 vs. open-VPS and LAVPS-NF) and shunt revisions. In the LAVPS-TPF group with abdominal history (*n* = 51), 12-month shunt failure rates (*P* = 0.020 vs. open-VS), repair frequency (*P* = 0.020 vs. open-VS), and abdominal complications (*P* = 0.003 and 0.006 vs. open-VS and LAVPS-NF) were reduced. In the LAVPS-TPF group with GCS scores of 13–15 (*n* = 152), shunt failure rates at 12 months, abdominal complications, and revision frequency were decreased (*P *< 0.05 vs. other groups). Compared to the LAVPS-NF group, neurological complications were also reduced (*P *= 0.001). Among revision surgeries (*n* = 28), fixed shunts resulted in improved shunt survival rates at 12 months, reduced abdominal complications, and secondary revisions (*P* < 0.05). Moreover, a more optimal recovery without neurological sequelae was achieved by shunt fixation than that by LAVPS-NF (*P* < 0.01).

**Conclusions:**

LAVPS-TPF significantly improved shunt survival rates at 12 months and reduced the incidence of abdominal shunt-related complications compared to open-VPS and LAVPS-NF, especially in patients with history of abdominal surgery, higher GCS scores, and revision surgeries. However, further studies are required to confirm these benefits.

## 1. Introduction

The ventriculoperitoneal shunt (VPS) has been widely used to treat hydrocephalus (HC) by redirecting accumulated cerebrospinal fluid (CSF) from the lateral ventricles to the abdominal cavity (1). However, abdominal complications may result in shunt failure, intracranial infection, and even death (2). The laparoscopy-assisted VPS (LAVPS) has been suggested as a safer and more effective method with lower complication rates (3, 4). Nonetheless, some patients experienced a recurrence of shunt-related abdominal complications shortly after surgery, leading to disappointing outcomes. Thus, to prevent abdominal symptoms and shunt dysfunction, we developed a novel method by fixing the abdominal catheter at the liver's falciform (FL) and round ligaments (RL) (two points). In this retrospective study, clinical records of adult patients with HC were analyzed, and outcomes after conventional laparotomy (open-VPS), LAVPS with no fixation (LAVPS-NF), and LAVPS with two-point fixation (LAVPS-TPF) were compared.

## 2. Methods

### 2.1. Patient population

**Figure 1:**
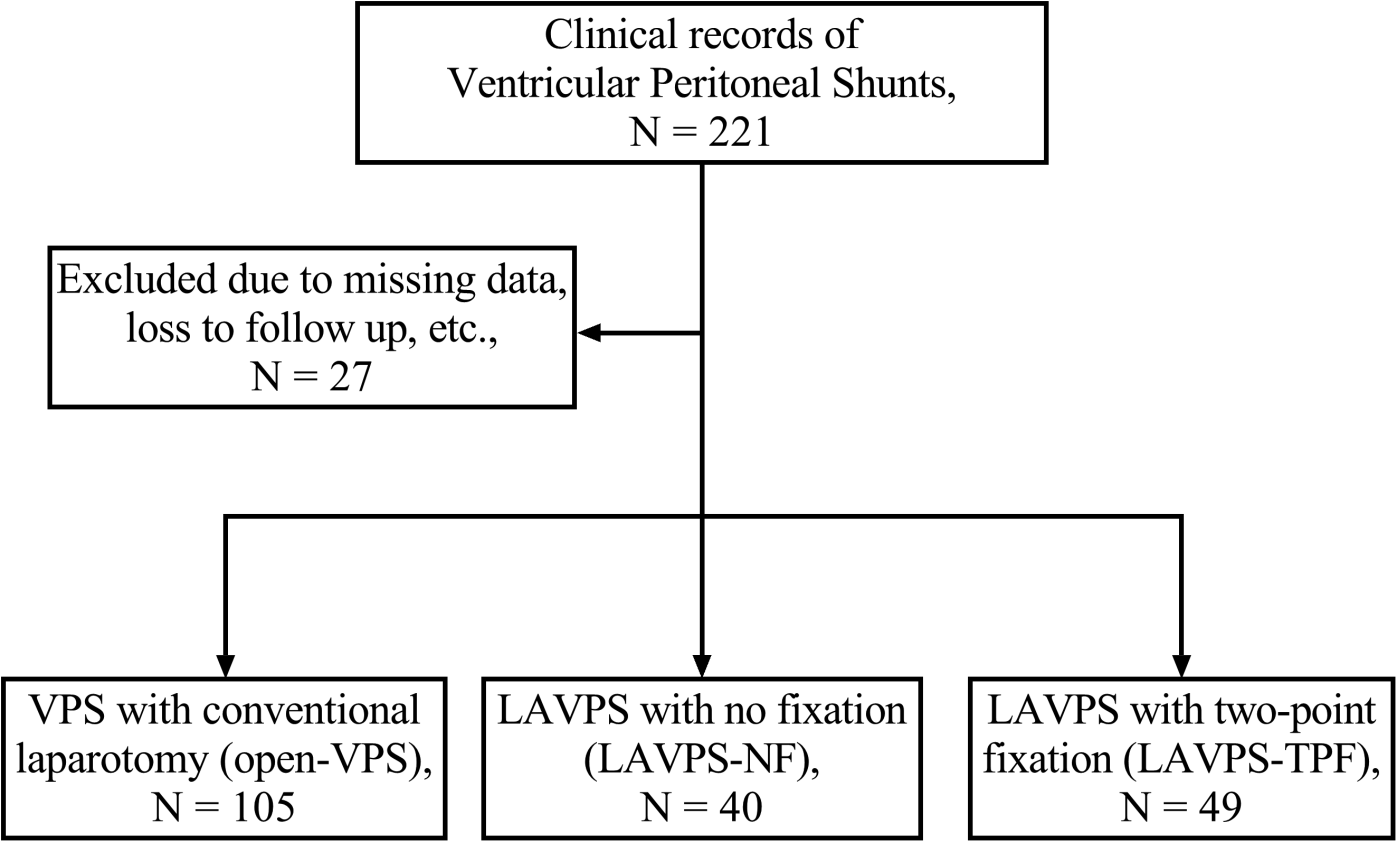
Diagram of the patient selection flow. N, clinical records in each group.

A total of 170 clinical records of 167 patients with HC who underwent either open-VPS, LAVPS-NF, or LAVPS-TPF between January 2015 and August 2020 at a single institution (selection flow in Figure 1) were analyzed. The inclusion and exclusion criteria are presented in the Supplementary Material.

The following data were recorded: patients’ age, sex, body mass index (BMI), signs and symptoms, Glasgow coma scale (GCS) scores, radiographic images, medical history, and surgical history. Based on their GCS scores at the time of admission, patients were categorized into three severity levels: mild (GCS 13–15), moderate (GCS 9–12), and severe (GCS 3–8) (5). In addition, patients were classified into communicating HC, obstructive HC, normal pressure HC (NPH), congenital HC, and acquired HC, based on their medical information. The American Society of Anesthesiologists (ASA) physical status scores evaluated by an anesthetist prior to surgery, post-surgery analgesia treatments, and complications were documented. Furthermore, post-surgical symptoms and radiographic outcomes were monitored, and modified Rankin scores (mRS) were assessed during follow-up (6).

### 2.2. Surgical procedures

**Figure 2:**
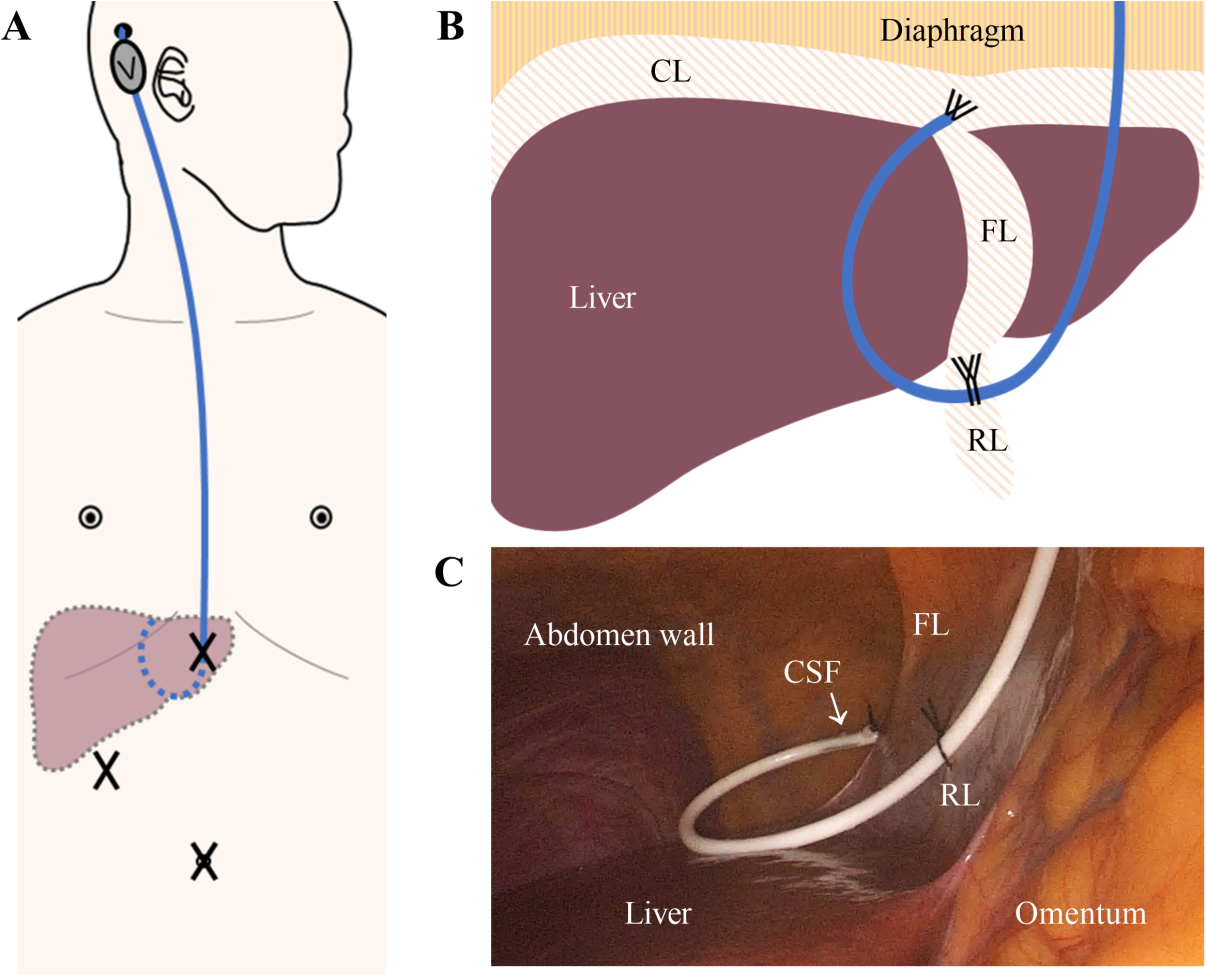
The two-point fixation technique. (**A**) Schematic diagram of the shunt (blue) subcutaneous pathway from the head to the abdomen. The subcutaneous VPS valve (V) behind the ear is connected to the ventricle and abdominal catheters. The abdominal catheter enters the abdominal cavity through the upper part of the abdominal trocars (X). Dashed lines indicate the intra-abdominal part of the shunt and liver. (**B**) Diagram of Two-Point Fixation to fix the catheter (blue) at livers’ FL and RL. (**C**) Interoperating laparoscopic photograph of the fixed catheter with clear, smoothly draining CSF. CL, coronary ligament. FL, falciform ligament. RL, round ligament. CSF, cerebrospinal fluid.

The surgical approach was determined in discussions between senior neurosurgeons. In the open-VPS group, the draining abdominal catheter was placed into the peritoneal cavity. For both LAVPS groups, the abdominal catheter was positioned on the liver's septal surface by the gastrointestinal surgeons. The shunt latency with clear CSF drips was verified under laparoscopy before closing. Particularly, in the LAVPS-TPF group, silk was used to suture the catheter tip on the falciform ligament, and the catheter body was tied to the round ligament as the second point (Figure 2). Details and short videos are presented in the Supplementary Material.

### 2.3. Outcomes

The rates of shunt failures diagnosed by a neurosurgeon at 6 and 12 months were the primary outcomes. Shunt (mostly valve) dysfunctions requiring either medicine or revision surgery were included. Secondary outcomes included shunt complications (i.e., obstruction, infection, cyst, over-drainage, and dislocation, among others), neurological complications (i.e., headache with or without vomiting, dizziness, unsteady gait and epilepsy among others), satisfaction regarding recovery (mRS = 0 or 1) (6, 7), and frequency of revision surgeries. All patients were followed up for at least 1 year.

### 2.4. Statistics

IBM SPSS Statistics (version 26.0, New York, United States) and GraphPad Prism (version 8.0, California, United States) were used for data analysis. Chi-square tests, Fisher's exact tests, Student's *t*-test, and analysis of variance (ANOVA) tests were applied, with continuous variables expressed as mean ± standard deviation (SD). Survival Kaplan–Meier graphs were generated with log-rank tests performed. Statistical significance was considered when *P *< 0.05.

## 3. Results

A total of 190 patient records with 105 open-VPS, 40 LAVPS-NF, and 49 LAVPS-TPF were reviewed (Table 1), including 28 revision surgeries. Sex, age, and BMI variances were found homogeneous in all three groups. Based on their GCS scores, patients were categorized into mild (*n* = 152), moderate (*n* = 27), and severe (*n* = 15) groups. Emergency surgery was performed on 21 patients (10 open-VPS, 2 LAVPS-NF, and 9 LAVPS-TPF).

**Table 1:** Overall patient population and outcomes after open-VPS, LAVPS-NF, and LAVPS-TFP.

Groups	Open-VPS	LAVPS-NF	LAVPS-TPF	*P* value	*P* value	*P* value
(Total = 194)	(*n* = 105)	(*n* = 40)	(*n* = 49)	Open-VPS vs. LAVPS-NF	Open-VPS vs. LAVPS-TPF	LAVPS-NF vs. LAVPS-TPF
Age	48.4 ± 15.09	54.6 ± 15.62	49.57 ± 14.79	0.073	0.887	0.273
Gender				0.094	0.887	0.104
Male	57	28	26			
Female	48	12	23			
BMI	23.6 ± 3.22	23.6 ± 2.66	24.4 ± 3.35	0.999	0.257	0.385
Hydrocephalus Etiology						
Communicating	66	25	44	0.968	0.001*	0.002*
Obstructive	32	15	5	0.419	0.006*	0.002*
Hyper pressure	71	24	41	0.388	0.037*	0.012*
Normal pressure	32	16	8	0.276	0.062	0.012*
Congenital	33	20	21	0.038*	0.166	0.501
Brain tumor	25	5	8	0.133	0.292	0.611
Post-ICH	22	8	12	0.899	0.622	0.614
Post-TBI	20	6	8	0.570	0.683	0.864
Other	5	1	0	>0.999	0.179	0.449
Abdominal surgery history	19	13	19	0.062	0.006*	0.539
Severity						
Mild (GCS 13–15)	79	34	39	0.205	0.552	0.509
Moderate (GCS 9–12)	18	2	7	0.058	0.654	0.178
Severe (GCS 3–8)	8	4	3	0.737	>0.999	0.696
ASA score				0.433	0.905	0.534
1	6	0	2			
2	55	21	24			
3	39	16	21			
4	5	3	2			
Revision surgery	8	8	12	0.042*	0.004*	0.614
Emergency surgery	10	2	9	0.377	0.120	0.102
Operation time	80.2 ± 13.13	79.7 ± 7.79	81.8 ± 9.65	>0.999	0.232	0.632
Bleeding volume	46.8 ± 66.78	30.8 ± 27.26	27.0 ± 34.52	0.246	0.088	0.944
In-hospital days	11.5 ± 4.85	11.0 ± 1.73	13.4 ± 4.31	0.767	0.038*	0.026*
Analgesia over 5 days	17	7	6	0.850	0.522	0.485
Shunt failure	19	9	1	0.548	0.006*	0.004*
Failure in 6 months	9	4	1	0.753	0.171	0.170
Failure in 12 months	15	5	1	0.780	0.020*	0.086
Shunt complications	20	9	1	0.642	0.004*	0.004*
Obstruction	10	5	0	0.599	0.031*	0.016*
Infection	6	1	1	0.674	0.308	>0.999
Pseudocyst	2	3	0	0.129	>0.999	0.087
Over-drainage	1	1	0	0.477	>0.999	0.449
Dislocation	1	0	0	>0.999	>0.999	–
Revision surgery post-VPS	15	6	1	>0.999	0.020*	0.042*
Follow-up days	914.2 ± 717.63	424.4 ± 196.44	518.9 ± 142.75	<0.001*	<0.001*	0.692
Abdominal pain	10	2	2	0.512	0.340	>0.999
Neurological complications	16	13	1	0.020*	0.015*	<0.001*
Headache/vomiting	6	9	0	0.006*	0.178	<0.001*
Dizzy	4	4	0	0.216	0.307	0.037*
Unsteady gait	4	5	0	0.115	0.307	0.016*
Epilepsy	2	0	1	>0.999	>0.999	>0.999
Death	6	3	2	0.707	>0.999	0.654
Satisfied_mRS	69	28	40	0.624	0.057	0.199

TBI, traumatic brain injury; ICH, intracranial hemorrhage.

* *P* < 0.05.

*χ*^2^ tests, Fisher's exact tests, and ANOVA tests were performed. Continuous variables are presented as mean ± SD. Satisfied_mRS: mRS = 0 or 1 at follow-up.

**Figure 3:**
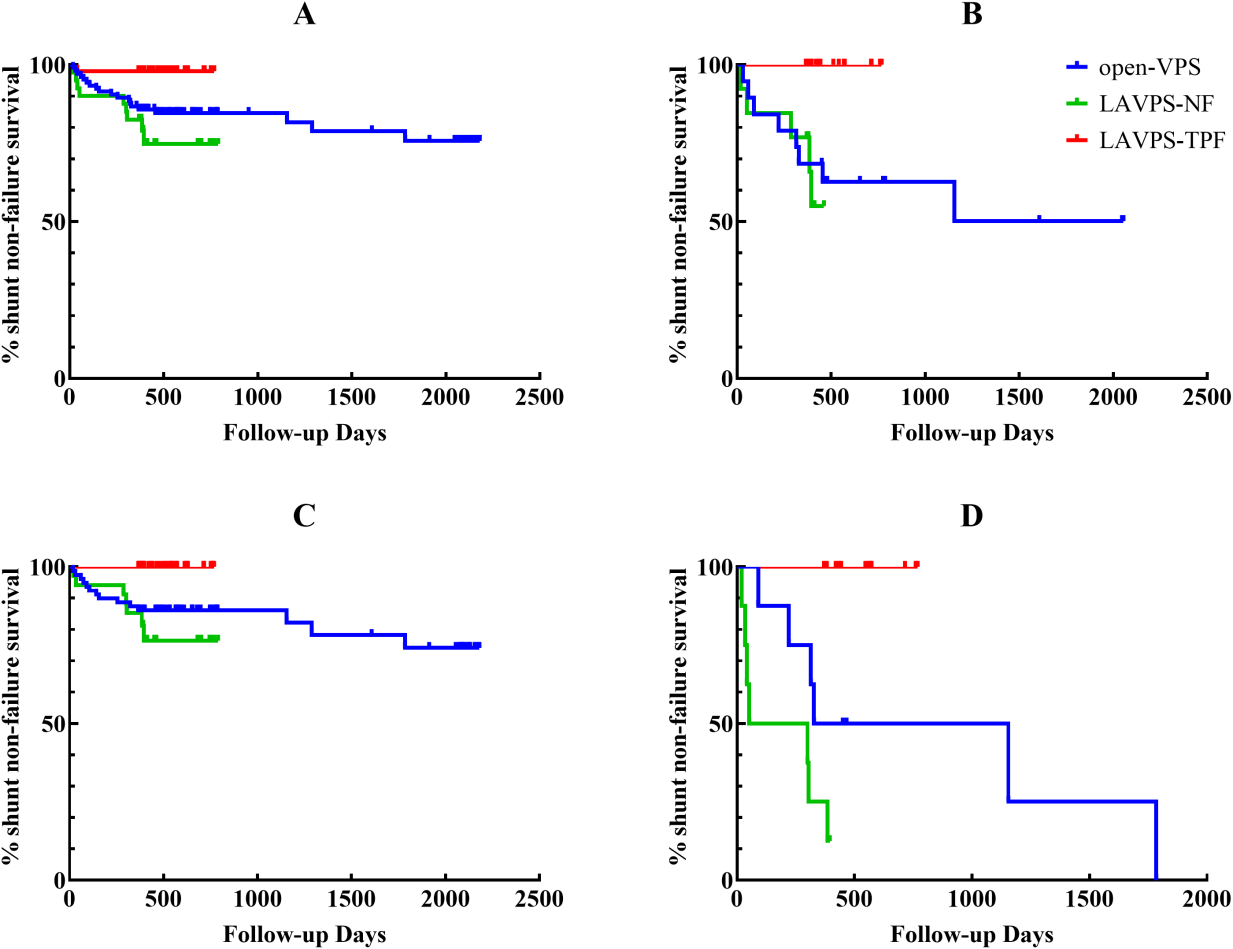
Survival curves of shunts in all patients (**A**, *P *= 0.045), patients with history of abdominal surgeries history (**B**, *P *= 0.010), patients with Glasgow coma scale score of 13–15 (**C**, *P *= 0.019), and revision surgeries (**D**, *P *= 0.008). Open-VPS (blue), ventriculoperitoneal shunt with conventional laparotomy; LAVPS-NF (green), laparoscopy-assisted ventriculoperitoneal shunt with no fixation; LAVPS-TPF (red), LAVPS with two-point fixation.

A total of 30 abdominal catheter issues were recorded after the surgeries (20 in open-VPS, nine in LAVPS-NF, and one in LAVPS-TPF). Compared with those in open-VPS, shunt failure rates at 12 months (2.04% vs. 14.29%, *P* = 0.020), abdominal catheter-related complications (*P* = 0.004), and neurological complications (*P* = 0.015) were reduced in LAVPS-TPF. Although no statistical significance was found compared with the shunt failure rate at 12 months in LAVPS-NF (12.50%, *P* = 0.086), abdominal and neurological complications (*P* = 0.004 and <0.001) declined in LAVPS-TPF. However, no significant differences were observed between the LAVPS-NT and open-VPS groups for the 12-month shunt survival (*P *= 0.780) and abdominal shunt-related complications (*P* = 0.642). And the shunt non-failure survival curves were presented with statistical differences (*P* = 0.0134, Figure 3A). Moreover, only one revision surgery was performed in LAVPS-TPF, which was statistically lesser than open-VPS (*P* = 0.020) and LAVPS-NF (*P* = 0.042). After surgery, pain management lasted for more than 5 days in 30 patients (17 in open-VPS, 7 in LAVPS-NF, and 6 in LAVPS-TPF; *P* > 0.05). A total of 14 patients experienced abdominal pain after hospital discharge (10 in open-VPS, 2 in LAVPS-NF, and 2 in LAVPS-TPF; *P* > 0.05). In addition, 13 patients experienced headaches (6 in open-VPS, 9 in LAVPS-NF, and none in LAVPS-TPF; *P* < 0.001 between the two LAVPS groups). Additionally, dizziness (*n* = 8, *P* = 0.037 LAVPS-NF vs. LAVPS-TPF), unsteady gait (*n* = 9, *P* = 0.016 LAVPS-NF vs. LAVPS-TPF), and epilepsy (*n* = 3, *P* > 0.999) were reported. During follow-up, 137 patients recovered well with mRS = 0 or 1 (69 in open-VPS, 28 in LAVPS-NF, and 40 in LAVPS-TPF; *P* > 0.05). However, 11 patients died during follow-up (reasons for deaths included shunt construction and/or infection, brain tumor, intracranial hemorrhage, pneumonia, traumatic brain injury-related multiorgan functional deficit, and unknown causes).

A total of 51 patients with history of abdominal surgeries (19 open-VPS, 13 LAVPS-NF, and 19 LAVPS-TPF; Table 2) were examined, and shunt failure was documented in 13 cases (eight open-VPS and five LAVPS-NF, *P* = 0.003 and 0.006 for LAVPS-TPF vs. open-VPS and LAVPS-NF). Among them, six cases were reported in the open-VPS group and three cases in the LAVPS-NF group at 12 months post-surgery, while no issues were reported in the LAVPS-TPF group (*P* = 0.020 and 0.058 vs. open-VPS and LAVPS-NF groups, respectively). Shunt failure rates decreased in the LAVPS-TPF group (*P* = 0.0145, Figure 3B). Among them, six and three shunt revisions were performed in the open-VPS (*P* = 0.020 vs. LAVPS-TPF) and LAVPS-NF groups (*P* = 0.058 vs. LAVPS-TPF), respectively. After surgery, seven patients required analgesia for more than 5 days, and four patients reported persistence of abdominal pain after discharge. During follow-up, eight cases reported neurological complications with no significant differences (*P* > 0.05). A total of 13, six, and 16 patients of the open-VPS, LAVPS-NF, and LAVPS-TPF groups were satisfied with their recovery (*P* = 0.049 between the two LAVPS groups).

**Table 2:** Outcomes of patients with abdominal history after open-VPS, LAVPS-NF, and LAVPS-TFP.

Groups	Open-VPS	LAVPS-NF	LAVPS-TPF	*P* value	*P* value	*P* value
(Total = 51)	(*n* = 19)	(*n* = 13)	(*n* = 19)	Open-VPS vs LAVPS-NF	Open-VPS vs LAVPS-TPF	LAVPS-NF vs LAVPS-TPF
Operation time	81.3 ± 12.17	82.2 ± 9.65	86.0 ± 8.96	>0.999	0.409	0.413
Bleeding volume	66.6 ± 132.76	39.2 ± 37.52	27.9 ± 31.81	0.477	0.225	0.365
In-hospital days	11.3 ± 2.63	11.1 ± 2.47	11.1 ± 3.16	0.797	0.825	0.979
Analgesia over 5 days	2	2	3	>0.999	>0.999	>0.999
Shunt failure	8	5	0	>0.999	0.003*	0.006*
Failure in 6 months	3	2	0	>0.999	0.230	0.157
Failure in 12 months	6	3	0	0.704	0.020*	0.058
Shunt complications	8	5	0	0.837	0.003*	0.006*
Obstruction	4	3	0	>0.999	0.105	0.058
Infection	3	1	0	0.629	0.230	0.406
Pseudocyst	0	1	0	0.406	—	0.406
Over-drainage	0	0	0	—	—	—
Dislocation	1	0	0	>0.999	>0.999	—
Revision surgery post-VPS	6	3	0	0.704	0.020*	0.058
Follow-up days	763.4 ± 685.98	333.6 ± 137.63	500.2 ± 143.13	0.034*	0.110	0.003*
Abdominal pain	2	0	2	0.502	>0.999	0.502
Neurological complications	3	4	1	0.401	0.604	0.132
Headache/vomiting	1	3	0	0.279	>0.999	0.058
Dizzy	0	1	0	0.406	—	0.406
Unsteady gait	2	1	0	>0.999	0.486	0.406
Epilepsy	0	0	1	—	>0.999	>0.999
Death	0	3	0	0.058	—	0.058
Satisfied_mRS	13	6	16	0.281	0.447	0.049*

* *P *< 0.05.

*χ*^2^ tests, Fisher's exact tests, and ANOVA tests were performed. Continuous variables are presented as mean ± SD. Satisfied_mRS: mRS = 0 or 1 at follow-up.

Then, patients of different GCS levels were investigated. Among 152 patients under mild level (79 open-VPS, 34 LAVPS-NF, and 39 LAVPS-TPF; Table 3), shunt failure occurred in 14, 7, and no patients of the open-VPS, LAVPS-NF (*P* = 0.694 vs. open-VPS), and LAVPS-TPF groups (*P* = 0.004 vs. open-VPS; *P* = 0.003 vs. LAVPS-NF), respectively. In the first 12 months after VPS, shunt failure occurred in 11 patients of the open-VPS group (*P* = 0.015 vs. LAVPS-TPF) and 4 patients of the LAVPS-NF group (*P* = 0.043 vs. LAVPS-TPF). The statistical difference among the three groups was observed in the shunt non-failure survival curve analysis (*P* = 0.021, Figure 3C). After open-VPS surgery, 13 shunt-related complications (i.e., eight obstructions, three infections, and two pseudocysts) were recorded, with 7 records in the LAVPS-NF group (*P* = 0.598 vs. open-VPS) and none in the LAVPS-TPF group (*P* = 0.005 vs. open-VPS; *P* = 0.003 vs. LAVPS-NF). Nine open-VPS and four LAVPS-NF were repaired thereafter with statistical significance (*P* = 0.029 and 0.043, respectively) compared to LAVPS-NPF (*n* = 0). A total of 24 patients still required analgesia after 5 days post-surgery (*P* > 0.05), and 12 patients had abdominal pain after discharge (*P* > 0.05 between the three groups). During follow-up, only one case with neurological complications (i.e., epilepsy) was recorded in the LAVPS-TPF, with *P* = 0.301 (vs. open-VPS, n = 12) and *P* = 0.001 (vs. LAVPS-NF, *n* = 10). Among them, four patients and seven patients manifested headaches after open-VPS (*P* = 0.017 vs. LAVPS-TPF) and LAVPS-NF (*P* = 0.003 vs. LAVPS-TPF), respectively; four patients had headaches after open-VPS (*P* > 0.999 vs. LAVPS-TPF) and LAVPS-NF (*P* = 0.043 vs. LAVPS-TPF). By the end of the follow-up, 120 patients recovered well (58 open-VPS, 27 LAVPS-NF, and 35 LAVPS-TPF; *P* > 0.05); however, five patients (four open-VPS and one LAVPS-NF) died.

**Table 3:** Outcomes of patients with GCS 13–15 after open-VPS, LAVPS-NF, and LAVPS-TFP.

Groups	Open-VPS	LAVPS-NF	LAVPS-TPF	*P* value	*P* value	*P* value
(Total = 152)	(*n* = 79)	(*n* = 34)	(*n* = 39)	Open-VPS vs LAVPS-NF	Open-VPS vs LAVPS-TPF	LAVPS-NF vs LAVPS-TPF
Operation time	80.2 ± 13.77	78.8 ± 6.45	81.6 ± 9.49	>0.999	0.1544	0.386
Bleeding volume	48.7 ± 74.09	24.4 ± 19.65	30.1 ± 38.10	0.063	0.144	0.433
In-hospital days	11 ± 3.87	10.7 ± 1.38	12.8 ± 4.55	0.641	0.030*	0.012*
Analgesia over 5 days	14	5	5	0.694	0.496	>0.999
Shunt failure	14	7	0	0.719	0.004*	0.003*
Failure in 6 months	8	2	0	0.721	0.051	0.213
Failure in 12 months	11	4	0	>0.999	0.015*	0.043*
Shunt complications	13	7	0	0.598	0.005*	0.003*
Obstruction	8	4	0	0.751	0.051	0.043*
Infection	3	0	0	0.553	0.550	—
Pseudocyst	2	2	0	0.582	>0.999	0.213
Over-drainage	0	1	0	0.301	—	0.466
Revision surgery post-VPS	9	4	0	>0.999	0.029*	0.043*
Follow-up days	912.3 ± 708.13	451.6 ± 189.68	529.0 ± 127.90	>0.999	0.001*	0.042*
Abdominal pain	8	2	2	0.721	0.494	>0.999
Neurological complications	12	10	1	0.080	0.301	0.001*
Headache/vomiting	4	7	0	0.017*	0.301	0.003*
Dizzy	4	4	0	0.239	>0.999	0.043*
Unsteady gait	2	3	0	0.159	>0.999	0.096
Epilepsy	2	0	1	>0.999	>0.999	>0.999
Death	4	1	0	>0.999	0.301	0.466
Satisfied_mRS	58	27	35	0.498	0.041*	0.218

* *P *< 0.05.

*χ*^2^ tests, Fisher's exact tests, and ANOVA tests were performed. Continuous variables are presented as mean ± SD. Satisfied_mRS: mRS = 0 or 1 at follow-up.

However, no significant differences were detected between the open-VPS (*n* = 18) and LAVPS-TPF (*n* = 7) groups of the moderate-level patients (Supplementary Table S1). Owing to the limited number of patients in the severe-level subgroup (eight open-VPS, four LAVPS-NF, and three LAVPS-TPF; Supplementary Table S2), statistical analysis was not performed.

A total of 28 revision surgery records (eight open-VPS, eight LAVPS-NF, and 12 LAVPS-TPF) were investigated (Table 4). Six shunts failed after open-VPS surgery (four in the first 12 months) with five repair surgeries, while seven shunts failed with five repair surgeries after LAVPS-NF (*P* > 0.999). Conversely, no shunt dysfunction or complication occurred in the LAVPS-TPF group (*P* = 0.001 vs. open-VPS; *P* < 0.001 vs. LAVPS-NF). The 12-month functional shunt rates were improved in the LAVPS-TPF groups (*P* = 0.014). The shunt failure rates declined in the LAVPS-TPF groups (*P < *0.001, Figure 3D). Unsteady gait and headaches persisted after two open-VPS and six LAVPS-NF surgeries, respectively. No patients had neurological symptoms after LAVPS-TPF surgeries (*P* = 0.001 vs. LAVPS-NF). Post-surgery analgesia treatment lasted for more than 5 days in four patients in the three groups (*P *> 0.05), and abdominal pain occurred in two patients in the open-VPS group (*P* > 0.05). At the end of the follow-up, optimal mRS scores were noted after 7 open-VPS, 1 LAVPS-NP, and 12 LAVPS-TPF surgeries (*P *< 0.001 between the LAVPS-NP and LAVPS-TPF).

**Table 4:** Outcomes after revision surgeries with open-VPS, LAVPS-NF, and LAVPS-TFP.

Groups	Open-VPS	LAVPS-NF	LAVPS-TPF	*P* value	*P* value	*P* value
(Total = 28)	(*n* = 8)	(*n* = 8)	(*n* = 12)	Open-VPS vs LAVPS-NF	Open-VPS vs LAVPS-TPF	LAVPS-NF vs LAVPS-TPF
Operation time	83.4 ± 9.13	80.4 ± 4.60	85.0 ± 12.69	>0.999	>0.999	>0.999
Bleeding volume	35.0 ± 18.52	30.0 ± 31.17	31.7 ± 33.46	0.702	0.801	0.912
In-hospital days	10.3 ± 1.04	10.6 ± 1.85	10.7 ± 1.97	0.624	0.591	0.963
Analgesia over 5 days	1	1	2	>0.999	>0.999	>0.999
Shunt failure	6	7	0	>0.999	0.001*	<0.001*
Failure in 6 months	1	4	0	0.282	0.400	0.014*
Failure in 12 months	4	4	0	>0.999	0.014*	0.014*
Shunt complications	6	7	0	>0.999	0.001*	<0.001*
Obstruction	4	3	0	>0.999	0.014*	0.049*
Infection	1	1	0	>0.999	0.400	0.400
Pseudocyst	0	3	0	0.200	—	0.049*
Over-drainage	0	1	0	>0.999	—	0.400
Dislocation	1	0	0	>0.999	0.400	–
Revision surgery post-VPS	5	5	0	>0.999	0.004*	0.004*
Follow-up days	601.1 ± 574.37	191.6 ± 168.84	544.8 ± 142.24	0.073	0.746	<0.001*
Abdominal pain	2	0	0	0.467	0.147	—
Neurological complications	2	6	0	0.132	0.147	0.001*
Headache/vomiting	0	6	0	0.007*	—	0.001*
Dizzy	0	1	0	>0.999	—	0.400
Unsteady gait	2	2	0	>0.999	0.147	0.147
Death	0	1	0	>0.999	—	0.400
Satisfied_mRS	7	1	12	0.010*	0.400	<0.001*

* *P *< 0.05.

*χ*^2^ tests, Fisher's exact tests, and ANOVA tests were performed. Continuous variables are presented as mean ± SD. Satisfied_mRS: mRS = 0 or 1 at follow-up.

Shunt survival and outcomes of patients with NPH (32 open-VPS, 16 LAVPS-NF, and 8 LAVPS-TPF), the elderly (over 60 years), and patients with BMI > 24 were also analyzed with no significant difference (tables in Supplementary Material).

## 4. Discussion

Since the first application of laparoscopy in HC by Dr. Rodgers in 1978, LAVPS has been suggested as a safe and effective approach in reducing the incidence of postoperative infection and complications compared to small-incision laparotomy VPS (3, 4, 8–10). The most significant advantage of LAVPS is the minimally invasive surgery with visualization that allows the accurate placement of the catheter (11–14). Different studies have demonstrated that the failure rate of laparoscopic surgery is lower (14.1%–15%) than that of laparotomy surgeries (16.9%–18.3%) (11, 15, 16). Additionally, the 30-day revision rate was significantly reduced in laparoscopic surgery (0%–1.2%), while secondary surgery rates at 6 and 12 months were not different (17, 18). In our study, the 12-month shunt failure rates were lowered by LAVPS-TPF (2.04%) rather than LAVPS-NF (12.50%) compared to open-VPS (14.29%), along with revision and complication rates.

A specific position for fixing the shunt requires further exploration. Some neurosurgeons believe that the catheter will wiggle into the pelvis with intestinal peristalsis (11, 19). However, distal shunt obstructions could be resolved by adjusting the catheter position under laparoscopy (20). In a retrospective study with 810 cases, Naftel et al. (4) concluded that blind placement of catheters in open-VPS might cause mistakes and increase the incidence of catheter distal obstruction (35.7%), which was reduced by LAVPS (4.8%). Rigante et al. (14) concur that shunt obstruction was associated with catheter position of the catheter. Thus, fixation could reduce catheter-related complications.

More studies revealed that the hepatic septal space, which is the highest position of the abdominal cavity in both supine and sitting positions, is ideal for the abdominal shunt to prevent omental wrapping and organ damage (21, 22). Svoboda et al. (23) placed the catheter through a falciform ligament defect, which reduced migration and obstruction in idiopathic NPH (iNPH). During treatment in 36 patients, Wang et al. (24) placed the catheter into the right subphrenic space through a hole of the sickle ligament with no complications during follow-up. Shao et al. (25) applied screws and vascular clips to anchor the catheter in the abdominal cavity or insert the catheter into the hepatic diaphragm through the falciform ligament. There were no catheter obstructions or infections during the 1-year follow-up. During our practice, after catheter placement at the liver's superoposterior surface, the catheter still migrated to a lower level as reported in a previous study (11, 19). To secure the distal tip safely, a silk suture was used to secure it at the falciform ligament. The highest position of both the falciform ligament and abdominal cavity, with a distance from the transition of the diaphragm and anterior abdominal wall, was determined as the fixing point. To prevent intractable hiccups, breathing issues (i.e., painful respiration and pneumothorax, among others), or pericardial injury, the distance is ∼2 cm to the diaphragm and could be adapted to anatomical variations (26).

To further enhance the benefits, a tight knot on the liver round ligament was employed to secure the catheter body on the liver septum. Additionally, the intra-abdominal region was limited to ∼15 cm to avoid a long curve reaching the omentum. In our study, infection was reported in one patient, but no patients had a distal catheter obstruction during follow-up in the LAVPS-TPF group.

The increased intracranial pressure (ICP) secondary to laparoscopic pneumoperitoneum has risks and causes temporary partial or complete shunt obstruction (27), which will immediately resolve after the removal of the laparoscope. An ICP of 25 cm H_2_O at a pneumoperitoneum pressure of 8–15 mmHg was considered safe (28, 29). The fixation procedure does not require a higher pressure or a longer period for pneumoperitoneum. In the present study, the pneumoperitoneum pressure was controlled at 12 mmHg, with the pneumoperitoneum time controlled for no more than 60 min (29). Releasing the pneumoperitoneum pressure slowly and confirming a clear CSF drainage before exiting the laparoscope are highly suggested.

Also, in our present study, LAVPS-TPF does not need additional operating time, in-hospital days, and pain treatments. Previous studies reported lesser blood loss, shorter operating time, faster recovery, and decreased analgesic use in LAVPS (4, 11–16, 18, 30). In our research, however, in-hospital days in LAVPS-TPF were longer than those in the open-VPS group. This could be attributed to more patients with history of abdominal surgery and revision surgeries in the LAVPS-TPF group, considering the non-significant results in those subgroups.

The most common rationale for shunt failure is the mechanical obstruction of the abdominal catheter, which can be significantly decreased by LAVPS (3.8%–4.8%) compared to open-VPS surgery (19.2%–35.7%) (4, 9, 31). Dislocation and displacement were also reduced in multiple studies (3, 32–34). With smaller incisions, LAVPS also reduced intra-abdominal infectious complications (0%–1.6% in LAVPS vs. 2.6%–5% in open-VPS) (9, 11, 15, 16, 35, 36). Regarding the different disease etiologies and conditions in patients enrolled in the present study, LAVPS-TPF did not improve the outcomes of all HC patients.

In patients with an abdominal surgery history, LAVPS has the advantage to deal with the common post-surgery intraperitoneal adhesions and avoid intra-abdominal injuries (37–39). This visual approach is excellent for revision surgeries to diagnose and address abdominal complications (e.g., peritoneal adhesions and CSF pseudocysts, among others, videos available in the Supplementary Material) (32, 40–42). Though LAVPS-NF did not statistically reveal advantages in patients with an abdominal history or revision surgeries in our study, LAVPS-TFP improved the shunt survival rates, prevented revision surgeries, and promoted better recovery after surgery. It was also consistent with our assumption that the two-point fixation technique protected shunts from the omentum.

Patients under mild levels were more likely to benefit from LAVPS-TPF instead of patients under severe levels, most of whom were acquired due to brain injuries (i.e., hemorrhage and trauma). Contrary to publications that showed LAVPS reduced complications and promoted cognitive and gait recovery in patients with NPH (4, 18, 30, 43), the advantage of LAVPS-TPF was not presented in our study compared to LAVPS-NF or open-VPS.

Patients with obesity would benefit from laparoscopic assistance by reducing the incidence of infections and revision surgeries, especially in those with BMI over 30 (39). However, in the present study, there was no difference between the outcomes after open-VPS and LAVPS surgeries for patients with BMI > 24 (Supplementary Table S5). Although the fixation seemed to reduce failure rates, shunt complications, and neurological complications compared with LAVPS-NF groups, it did not improve patients’ mRS scores and outcomes. The obviously limited population of patients with higher BMI and paucity of other obesity indices (e.g., abdominal circumference and waist-to-hip ratio) could be attributed to the above findings.

## 5. Limitations

As a retrospective study, the evidence level for the conclusions is relatively lower. In the present study, three senior neurosurgeons and two gastrointestinal surgeons participated in determining surgical approaches and performing surgical procedures, probably leading to inevitable systemic bias. Patients who underwent LAVPS-NF and LAVPS-TPF were limited. Future studies with larger sample sizes, randomized controlled designs, and multiple-center cooperation are required to further confirm the advantages of LAVPS-TPF. Complication-related outcomes were potentially biased, mostly from subjective symptoms such as pain, headache, and dizziness, which lacked objective evaluation in the present self-adjudicated study. Also, risk factors for complications, repair surgeries, and terrible outcomes warrant further investigation. Additionally, we only focused on adult patients with HC. Although pediatric patients can also benefit from LAVPS (44, 45), catheter fixation should be discussed carefully.

## 6. Conclusions

In this study, our data showed that LAVPS-TPF statistically reduced shunt failure rates and the incidence of abdominal shunt-related complications. This novel two-point fixation technique further enhanced the benefits of LAVPS, especially in patients with abdominal history, higher GCS scores, and repair surgeries. Additionally, LAVPS-TPF decreased the frequency of revision surgeries in patients with history of abdominal surgery and revision surgeries.

## Data Availability

The original contributions presented in the study are included in the article/**Supplementary Material**, further inquiries can be directed to the corresponding authors.
