# Supplementary material for: Two-point fixation enhanced the outcome of laparoscopy-assisted ventriculoperitoneal shunt in adult patients with hydrocephalus: a retrospective study
Source: Front Surg. 2023 Jul 13;10:1135818. doi: 10.3389/fsurg.2023.1135818 (PMC10390223; doi:10.3389/fsurg.2023.1135818)
Supplement: Supplementary file 1 [file Table1.docx]

Supplementary Material

Two-Point Fixation Enhanced the Outcome of Laparoscopy-Assisted Ventriculoperitoneal Shunt in Adult Patients with Hydrocephalus: A Retrospective Study

Jing-Nan Wu†, Yu-Jie Zhou†, Lei Wang, Jin-Lu Gan, Jian Wang, Hong-Yang Zhao^*^, De-Qiang Lei ^*^

† These authors contributed equally to this work and share first authorship

*** Correspondence:** De-Qiang Lei and Hong-Yang Zhao
Dr. De-Qiang Lei: [ldqtz@163.com](mailto:ldqtz@163.com)

Dr. Hong-Yang Zhao:[hyzhao750@sina.com](mailto:hyzhao750@sina.com)

# Inclusion and exclusion criteria

Neurosurgeons established the diagnosis of hydrocephalus (HC) according to clinical characteristics (i.e., headache, vomiting, unconsciousness, and coma among others.) and radiographic outcome with an Evans index greater than 0.3 (1). The indications for laparoscopic revision surgery include shunt obstruction caused by intraabdominal adhesions, catheter fracture or displacement, cerebrospinal fluid (CSF) pseudocyst, diagnostics, and conversion from the ventricular atrial shunt to ventricular shunt (2).

The inclusion criteria were as follows: 1) at least 18 years old; 2) newly diagnosed with HC that required ventriculoperitoneal shunt (VPS) surgery or post-surgery complications that required revision surgery; and 3) written informed consent. The exclusion criteria were as follows: 1) pregnancy; 2) less than 12 months of estimated survival time; 3) missing data or lost to follow-up; and 4) absence of a written informed consent.

# Surgical procedures

The surgeries were performed with the patient in the supine position with the head turned to the left (or right per patient’s condition). A straight incision was made at the puncture point of the right lateral ventricle (or left according to the patient’s condition) after skin disinfection. Next, a subcutaneous chamber was dissected caudally to the puncture point for the VPS valve. The upper abdominal skin was incised caudally to the xiphoid process on the midline. A subcutaneous tunnel was formed from the head to the upper abdomen through the neck and chest by a metal strip, in which the shunt tube was then guided from the abdomen to the head incision.

Skull drilling was performed while with the abdomen was concurrently being operated on, and a cross-shaped cut was employed in the dura. Hereafter, the ventricle catheter of the shunt was inserted into the lateral ventricle. After the CSF was confirmed to drain smoothly without bleeding, the ventricle tube, valve (the pressure was preset according to the CSF pressure measured prior to surgery), and abdominal tube were connected firmly and fixed with the shunt valve settled in the head subcutaneous chamber. Thereafter, the clear CSF was reconfirmed, draining from the abdominal end smoothly without blood.

For the **open-VPS group**, the abdominal incision was extended until 10 cm, and the peritoneal cavity was dissected. Then, the abdominal catheter of the shunt was gently inserted into the peritoneal cavity with an intraabdominal length of approximately 15 cm. Finally, the peritoneal cavity and skin incision were closed without damaging the catheter.

For **laparoscopy-assisted VPS (LAVPS) groups**, three trocars were set, a 12-mmHg pneumoperitoneum was induced, and the abdominal end of the shunt was inserted into the peritoneal cavity under laparoscopic guidance. The catheter end was placed on the septal surface of the liver with an intraabdominal length of approximately 15 cm.

In the **LAVPS with no fixation (LAVPS-NF) group**, the abdomen was then closed after clear CSF drips were confirmed.

In the **LAVPS with two-point fixation (LAVPS-TPF) group**, a taper pointed needle with a non-absorbable suture was passed through the tip of the multi-side-holed catheter; the tip was then fixed on the falciform ligament (*the first point*) approximately 2 cm to the lower edge of the diaphragm and 2 cm to the anterior abdominal wall. Next, the body of the catheter is wrapped around and knotted to the middle of the round ligament of the liver (*the second point*) using a silk suture to ensure the catheter’s abdominal end above the septal surface of the liver. The positions of the two points could be adapted to anatomical variations. Finally, the trocars were removed, and the incisions were closed after confirming shunt patency and absence of bleeding.

**References:**

1. Chen T, Ren Y, Wang C, Huang B, Lan Z, Liu W, et al. Risk factors for hydrocephalus following fourth ventricle tumor surgery: A retrospective analysis of 121 patients. PLoS One. 2020;15(11):e0241853.

2. Martin K, Baird R, Farmer JP, Emil S, Laberge JM, Shaw K, et al. The use of laparoscopy in ventriculoperitoneal shunt revisions. J Pediatr Surg. 2011;46(11):2146-50.

# Videos

**Video 01.** In this case of a revision surgery, a broken tube was found in the abdominal cavity and then removed. Afterwards, the tip of a new abdominal catheter (Medtronic Inc.) was sutured to the falciform ligament. And the catheter body was tied to the round ligament. Throughout the procedure, clear CSF drips were visible.

**Video 02.** In this case of another revision surgery, a CSF pseudocyst was diagnosed in the abdominal cavity. The drainage patency was obtained by removing the pseudocyst, and further secured by loosening peritoneal adhesions and fixing the tip to the falciform ligament.

**Video 03.** At the end of the fixation procedure, the patency of the CSF drainage and absence of bleeding were carefully confirmed before exiting the laparoscope.

# Supplemental Tables

**Supplemental Table 1. Outcomes of patients with GCS 9~12 after open-VPS, LAVPS-NF and LAVPS-TFP**

| Groups (Total = 27) | open-VPS (n = 18) | LAVPS-NF (n = 2) | LAVPS-TPF (n = 7) |
| --- | --- | --- | --- |
| Abdominal surgery history | 7 | 0 | 1 |
| Revision surgery | 5 | 0 | 1 |
| Operation time | 81.8 ± 11.42 | 76.5 ± 2.12 | 79.4 ± 5.86 |
| Bleeding volume | 34.44 ± 19.70 | 65.00 ± 49.50 | 14.29 ± 4.50 |
| In-hospital days | 12.67 ± 4.43 | 10.50 ± 2.12 | 16.00 ± 2.52 |
| Shunt failure | 4 | 0 | 1 |
| Failure in 6 months | 0 | 0 | 1 |
| Failure in 12 months | 3 | 0 | 1 |
| Shunt complications | 5 | 0 | 1 |
| Obstruction | 2 | 0 | 0 |
| Infection | 2 | 0 | 1 |
| Dislocation | 1 | 0 | 0 |
| Revision surgery post VPS | 5 | 0 | 1 |
| Analgesia over 5 days | 3 | 0 | 1 |
| Abdominal pain | 3 | 0 | 0 |
| Neurological complications | 3 | 1 | 0 |
| Headache/vomiting | 1 | 0 | 0 |
| Unsteady gait | 2 | 1 | 0 |
| Death | 0 | 0 | 1 |
| Satisfied_mRS | 11 | 1 | 5 |

Continuous variables are presented as mean ± standard deviation. Satisfied_mRS: mRS = 0 or 1 at follow-up.

**Supplemental Table 2. Outcomes of patients with GCS 3~8 after open-VPS, LAVPS-NF and LAVPS-TFP**

| Groups (Total = 15) | open-VPS (n = 8) | LAVPS-NF (n = 4) | LAVPS-TPF (n = 3) |
| --- | --- | --- | --- |
| Abdominal surgery history | 1 | 2 | 0 |
| Revision surgery | 0 | 3 | 0 |
| Operation time | 76.9 ± 10.64 | 88.8 ± 14.36 | 89.0 ± 16.52 |
| Bleeding volume | 55.0 ± 60.24 | 67.50 ± 39.48 | 16.67 ± 5.77 |
| In-hospital days | 13.9 ± 11.09 | 13.50 ± 2.65 | 14.33 ± 1.155 |
| Shunt failure | 1 | 2 | 0 |
| Failure in 6 months | 1 | 2 | 0 |
| Failure in 12 months | 1 | 2 | 0 |
| Shunt complications | 2 | 2 | 0 |
| Obstruction | 0 | 1 | 0 |
| Infection | 1 | 1 | 0 |
| Cyst | 0 | 1 | 0 |
| Over drainage | 1 | 0 | 0 |
| Revision surgery post VPS | 1 | 2 | 0 |
| Analgesia over 5 days | 0 | 0 | 0 |
| Abdominal pain | 2 | 0 | 0 |
| Neurological complications | 1 | 2 | 0 |
| Headache/vomiting | 1 | 2 | 0 |
| Unsteady | 0 | 1 | 0 |
| Death | 2 | 2 | 1 |
| Satisfied_mRS | 0 | 0 | 3 |

Continuous variables are presented as mean ± standard deviation. Satisfied_mRS: mRS = 0 or 1 at follow-up.

**Supplemental Table 3. Outcomes of patients with normal pressure hydrocephalus after open-VPS, LAVPS-NF and LAVPS-TFP**

| Groups (Total = 56) | open-VPS  (n = 32) | LAVPS-NF  (n = 16) | LAVPS-TPF  (n = 8) | *P* value  Open-VPS vs LAVPS-NF | *P* value  Open-VPS vs LAVPS-TPF | *P* value  LAVPS-NF vs LAVPS-TPF |
| --- | --- | --- | --- | --- | --- | --- |
| Abdominal surgery history | 4 | 10 | 4 | < 0.001* | 0.037* | 0.673 |
| Revision surgery | 2 | 5 | 4 | 0.033 | 0.010* | 0.412 |
| Operation time | 82.7 ± 18.61 | 81.4 ± 8.62 | 81.8 ± 7.44 | 0.963 | 0.988 | 0.999 |
| Bleeding volume | 51.7 ± 53.85 | 27.8 ± 30.27 | 17.5 ± 8.86 | 0.193 | 0.134 | 0.853 |
| In-hospital days | 12.3 ± 6.56 | 10.4 ± 2.125 | 12.8 ± 2.053 | 0.462 | 0.968 | 0.539 |
| Shunt failure | 5 | 4 | 0 | 0.457 | 0.563 | 0.262 |
| Failure in 6 months | 4 | 1 | 0 | 0.652 | 0.566 | > 0.999 |
| Failure in 12 months | 5 | 3 | 0 | > 0.999 | 0.563 | 0.526 |
| Shunt complications | 5 | 5 | 0 | 0.267 | 0.563 | 0.130 |
| Obstruction | 2 | 3 | 0 | 0.316 | > 0.999 | 0.526 |
| Infection | 2 | 0 | 0 | 0.546 | > 0.999 | - |
| Cyst | 1 | 2 | 0 | 0.254 | > 0.999 | 0.536 |
| Revision surgery post VPS | 3 | 3 | 0 | 0.386 | > 0.999 | 0.526 |
| Analgesia over 5 days | 6 | 4 | 0 | 0.712 | 0.318 | 0.262 |
| Abdominal pain | 3 | 0 | 0 | 0.541 | > 0.999 | - |
| Neurological complications | 6 | 4 | 0 | 0.712 | 0.318 | 0.262 |
| Headache/vomiting | 0 | 4 | 0 | 0.009* | - | 0.262 |
| Dizzy | 1 | 0 | 0 | > 0.999 | > 0.999 | - |
| Unsteady gait | 3 | 1 | 0 | > 0.999 | > 0.999 | > 0.999 |
| Epilepsy | 2 | 0 | 0 | 0.546 | > 0.999 | - |
| Death | 2 | 3 | 0 | 0.316 | > 0.999 | 0.526 |
| Satisfied_mRS | 21 | 10 | 8 | > 0.999 | 0.0803 | 0.066 |

*: *P* < 0.05. Fisher’s exact tests and ANOVA tests were performed. Continuous variables are presented as mean ± standard deviation. Satisfied_mRS: mRS = 0 or 1 at follow-up.

**Supplemental Table 4. Outcomes of older patients (age ≥ 60) after open-VPS, LAVPS-NF and LAVPS-TFP**

| Groups (Total = 68) | open-VPS  (n = 32) | LAVPS-NF  (n = 19) | LAVPS-TPF  (n = 17) | P value  Open-VPS vs LAVPS-NF | P value  Open-VPS vs LAVPS-TPF | P value  LAVPS-NF vs LAVPS-TPF |
| --- | --- | --- | --- | --- | --- | --- |
| Abdominal surgery history | 6 | 10 | 3 | 0.027* | > 0.999 | 0.041* |
| Revision surgery | 2 | 3 | 0 | 0.348 | 0.537 | 0.231 |
| Operation time | 76.7 ± 6.90 | 82.3 ± 9.86 | 79.2 ± 10.29 | 0.073 | 0.602 | 0.535 |
| Bleeding volume | 46.41 ± 101.7 | 36.32 ± 36.05 | 15.00 ± 5.000 | 0.882 | 0.328 | 0.657 |
| In-hospital days | 10.06 ± 3.537 | 10.58 ± 2.036 | 15.00 ± 4.569 | 0.867 | < 0.001* | 0.001* |
| Shunt failure | 3 | 3 | 0 | 0.659 | 0.542 | 0.231 |
| Failure in 6 months | 2 | 1 | 0 | > 0.999 | 0.537 | > 0.999 |
| Failure in 12 months | 2 | 1 | 0 | > 0.999 | 0.537 | > 0.999 |
| Shunt complications | 3 | 3 | 0 | 0.659 | 0.542 | 0.231 |
| Obstruction | 1 | 2 | 0 | 0.548 | > 0.999 | 0.487 |
| Infection | 2 | 1 | 0 | > 0.999 | 0.537 | > 0.999 |
| Revision surgery post VPS | 2 | 1 | 0 | > 0.999 | 0.537 | > 0.999 |
| Analgesia over 5 days | 3 | 6 | 2 | 0.063 | > 0.999 | 0.236 |
| Abdominal pain | 3 | 0 | 2 | 0.285 | > 0.999 | 0.216 |
| Neurological complications | 1 | 4 | 0 | 0.058 | > 0.999 | 0.106 |
| Headache/vomiting | 1 | 2 | 0 | 0.548 | > 0.999 | 0.487 |
| Dizzy | 0 | 1 | 0 | 0.373 | - | > 0.999 |
| Unsteady gait | 0 | 1 | 0 | 0.373 | - | > 0.999 |
| Death | 1 | 3 | 0 | 0.140 | > 0.999 | 0.231 |
| Satisfied_mRS | 23 | 13 | 15 | > 0.999 | 0.287 | 0.236 |

*: *P* < 0.05. Fisher’s exact tests and ANOVA tests were performed. Continuous variables are presented as mean ± standard deviation. Satisfied_mRS: mRS = 0 or 1 at follow-up.

**Supplemental Table 5. Outcomes of patients with BMI > 24 after open-VPS, LAVPS-NF and LAVPS-TFP**

| Groups (Total = 94) | open-VPS  (n = 42) | LAVPS-NF  (n = 21) | LAVPS-TPF  (n = 31) | P value  Open-VPS vs LAVPS-NF | P value  Open-VPS vs LAVPS-TPF | P value  LAVPS-NF vs LAVPS-TPF |
| --- | --- | --- | --- | --- | --- | --- |
| Abdominal surgery history | 7 | 6 | 12 | 0.329 | 0.057 | 0.558 |
| Revision surgery | 3 | 4 | 6 | 0.209 | 0.156 | > 0.999 |
| Operation time | 78.2 ± 9.63 | 77.5 ± 6.10 | 82.4 ± 10.88 | 0.962 | 0.144 | 0.162 |
| Bleeding volume | 38.81 ± 30.02 | 32.86 ± 26.53 | 33.55 ± 41.92 | 0.787 | 0.788 | 0.997 |
| In-hospital days | 11.62 ± 6.129 | 11.00 ± 1.378 | 13.32 ± 4.833 | 0.889 | 0.326 | 0.233 |
| Shunt failure | 7 | 5 | 1 | 0.513 | 0.127 | 0.034* |
| Failure in 6 months | 4 | 3 | 1 | 0.677 | 0.387 | 0.291 |
| Failure in 12 months | 7 | 3 | 1 | > 0.999 | 0.127 | 0.291 |
| Shunt complications | 5 | 5 | 1 | 0.280 | 0.232 | 0.034* |
| Obstruction | 1 | 3 | 0 | 0.104 | > 0.999 | 0.060 |
| Infection | 2 | 1 | 1 | > 0.999 | > 0.999 | > 0.999 |
| Cyst | 1 | 1 | 0 | > 0.999 | > 0.999 | 0.404 |
| Over drainage | 0 | 1 | 0 | 0.333 | - | 0.404 |
| Dislocation | 1 | 0 | 0 | > 0.999 | > 0.999 | - |
| Revision surgery post VPS | 5 | 4 | 1 | 0.466 | 0.232 | 0.145 |
| Analgesia over 5 days | 7 | 3 | 3 | > 0.999 | 0.502 | 0.675 |
| Abdominal pain | 7 | 1 | 1 | 0.251 | 0.127 | > 0.999 |
| Neurological complications | 11 | 8 | 0 | 0.389 | 0.002* | < 0.001* |
| Headache/vomiting | 5 | 6 | 0 | 0.158 | 0.068 | 0.003* |
| Dizzy | 3 | 3 | 0 | 0.391 | 0.257 | 0.060 |
| Unsteady gait | 3 | 4 | 0 | 0.209 | 0.257 | 0.022* |
| Death | 2 | 0 | 1 | 0.548 | > 0.999 | > 0.999 |
| Satisfied_mRS | 27 | 15 | 25 | 0.778 | 0.191 | 0.512 |

*: *P* < 0.05. Fisher’s exact tests and ANOVA were performed. Continuous variables are presented as mean ± standard deviation. Satisfied_mRS: mRS = 0 or 1 at follow-up.
